# *Drosophila* Embryos as Model Systems for Monitoring Bacterial Infection in Real Time

**DOI:** 10.1371/journal.ppat.1000518

**Published:** 2009-07-17

**Authors:** Isabella Vlisidou, Andrea J. Dowling, Iwan R. Evans, Nicholas Waterfield, Richard H. ffrench-Constant, Will Wood

**Affiliations:** 1 Department of Biology and Biochemistry, University of Bath, Bath, United Kingdom; 2 School of Biological Sciences, University of Exeter in Cornwall, Penryn, United Kingdom; Stanford University, United States of America

## Abstract

*Drosophila* embryos are well studied developmental microcosms that have been used extensively as models for early development and more recently wound repair. Here we extend this work by looking at embryos as model systems for following bacterial infection in real time. We examine the behaviour of injected pathogenic (*Photorhabdus asymbiotica*) and non-pathogenic (*Escherichia coli*) bacteria and their interaction with embryonic hemocytes using time-lapse confocal microscopy. We find that embryonic hemocytes both recognise and phagocytose injected wild type, non-pathogenic *E. coli* in a Dscam independent manner, proving that embryonic hemocytes are phagocytically competent. In contrast, injection of bacterial cells of the insect pathogen *Photorhabdus* leads to a rapid ‘freezing’ phenotype of the hemocytes associated with significant rearrangement of the actin cytoskeleton. This freezing phenotype can be phenocopied by either injection of the purified insecticidal toxin Makes Caterpillars Floppy 1 (Mcf1) or by recombinant *E. coli* expressing the *mcf1* gene. Mcf1 mediated hemocyte freezing is *shibire* dependent, suggesting that endocytosis is required for Mcf1 toxicity and can be modulated by dominant negative or constitutively active Rac expression, suggesting early and unexpected effects of Mcf1 on the actin cytoskeleton. Together these data show how *Drosophila* embryos can be used to track bacterial infection in real time and how mutant analysis can be used to genetically dissect the effects of specific bacterial virulence factors.

## Author Summary

The humble fruit fly has formed an important model for the study of bacterial infection both by insect specific and mammalian pathogens. However, many studies of bacterial infection rely upon death of the insect host, or actin cytoskeleton staining of specific host cells, at fixed end-points to look at infection or the mode of action of different bacterial toxins. Here, we use *Drosophila* embryos in a novel application to look at bacterial infection in real time. Contrary to popular belief, embryonic hemocytes both recognise and ingest injected *Escherichia coli*. This is a dynamic process in which the bacteria are recognised by, and adhere to, the phagocytes in a process that can be dramatically seen in real time using time-lapse confocal microscopy. In contrast, when cells of the insect pathogen *Photorhabdus* are introduced, the hemocytes become frozen and are unable to engulf the invading bacteria. Using both recombinant *E. coli* expressing the bacterial toxin Makes Caterpillars Floppy, and also purified toxin itself, we show how genetic mutants of *Drosophila* can be used to dissect the role of bacterial toxins in infection. Such approaches should provide a useful model in which to study infection by other pathogens and their associated toxins.

## Introduction

*Drosophila* is now widely established as a useful genetic model for looking at microbial infection with recent studies now encompassing both bacterial [1], viral [2] and even fungal infections [3]. Different *Drosophila* infection models have also begun to mimic different types of infections. For example, several groups are now developing systems in which to examine bacterial intestinal infections [4] as well as the more highly studied model of septic injury involving injection of bacteria directly into the open insect blood system or hemocoel.

Within each of these infection models, three different aspects of infection can be examined [5]. First, the innate immune response, the mechanism whereby the fly attempts to kill, isolate or neutralize the invading microbe. Second, microbial virulence, the mechanism whereby the invading microbe seeks to evade or overcome the host immune response. Finally, third, changes in host pathology that can relate either to adverse effects generated by the invading microbe or indeed the host immune response itself [5]. Whilst it is easy to recognize these three aspects of infection it is often harder to examine the interactions between them. It is possible, for example, to examine the effects of a recombinant bacterial toxin on infection, but it is more difficult to examine the role of specific virulence factors in neutralizing specific elements of the immune system, such as phagocytes. Consequently, despite the extensive and highly successful efforts of many researchers to develop infection models in *Drosophila*, the outcomes of infection are often measured by end-points such as insect death (survival of a cohort of insects over time) or changes in cell morphology at fixed periods throughout infection (often monitored by staining different elements of the cytoskeleton). Although this problem can, to some extent, be addressed by the use of reporter constructs (e.g. *Diptericin-LacZ*) that provide quantitative or visual read-outs from specific immune response genes, we still lack the ability to follow bacterial infections in real-time in the critical early stages of infection. We are therefore unable to visualise the outcome of the first interactions between insect phagocytes and invading microbes, interactions that will determine the future success of the infection itself. To address this need, here we use *Drosophila* embryos, specifically embryonic hemocytes, as models for studying the early stages of infection in real-time using time lapse confocal microscopy.

*Drosophila* embryos and their development are extremely well documented and recent attention has focused on the role of the embryonic hemocytes in early embryonic development. Embryonic hemocytes are highly motile macrophage-like cells that migrate throughout the developing embryo following stereotypical routes to disperse from their point of origin to eventually distribute themselves equally throughout the animal by late embryonic stages [6],[7]. During their migration hemocytes play many important roles in development including the phagocytic clearance of apoptotic cells within the embryo, as well as the production and secretion of many extracellular matrix proteins [8]. They are also essential for the proper development of many key structures such as the gut and Central nervous system [9].

Although these developmental roles are well documented it is less clear how competent these cells are to respond to infection and whether they play a significant role in the immune response of the embryo. Embryonic hemocytes lend themselves beautifully to live imaging studies since fluorescent probes can be expressed specifically in these cells using hemocyte specific promoters and their movements subsequently imaged within living embryos using confocal timelapse microscopy. *Drosophila* embryos therefore represent an easily injectable, containable and closed experimental system into which bacteria can be injected and their subsequent interactions with resident hemocytes observed in real time.

Many of the studies developing *Drosophila* as models for bacterial infections have used bacteria pathogenic to man [5]. Thus several studies have used *Pseudomonas aeruginosa*, *Salmonella typhimurium*, *Staphylococcus aureus* or *Vibrio cholera* to look at insects as models for mammalian infection [10]–[15]. However, the role of insect specific pathogens, or pathogens capable of infecting both insects and man, has been less well studied. We have been developing the Gram-negative bacterium *Photorhabdus asymbiotica* as a model system in which to study cross-talk between virulence factors developed against insects that can also be deployed against mammalian immune responses. *P. asymbiotica* strains have been recovered from human wounds [16] and are vectored by nematodes that usually invade and kill insects [17]. We have recently catalogued the full range of virulence factors that this bacteria has at its disposal for infecting insects and humans [18]. Anti-insect virulence in *Photorhabdus* bacteria is associated with the dominant toxin Makes Caterpillars Floppy 1 or Mcf1 [19]. This toxin causes extensive apoptotic cell death in both the midgut epithelium and circulating hemocytes of caterpillar hosts. Access to both purified Mcf1 toxin and recombinant *E. coli* expressing the *mcf1* gene makes this an ideal virulence factor in which to study early interactions between an insect pathogen and insect phagocytes.

Here, we pioneer the use of *Drosophila* embryos as models to study bacterial infection in real time. We show that embryonic hemocytes both recognise and phagocytose non-pathogenic *E. coli* in a Dscam independent manner. In contrast, we show that the cells of the insect pathogen *Photorhabdus* instantly freeze the highly mobile phagocytes. This freezing phenotype can be phenocopied either by injection of recombinant *E. coli* expressing the *mcf1* gene, or by injection of the purified toxin itself. The ability to image these first early stages of infection therefore facilitates a direct examination of the Mcf1 virulence factor neutralizing the phagocytic role of the embryonic hemocytes. Moreover, examination of the role of Mcf1 can be dissected genetically using mutants that either interfere with its endocytosis into target cells, or Rac signalling mutants that hint at early and unexpected Mcf1 mediated effects on the phagocyte cytoskeleton.

## Results

### Specific binding of non pathogenic *E. coli* by stage 15 *D. melanogaster* embryonic hemocytes

**Figure 1:**
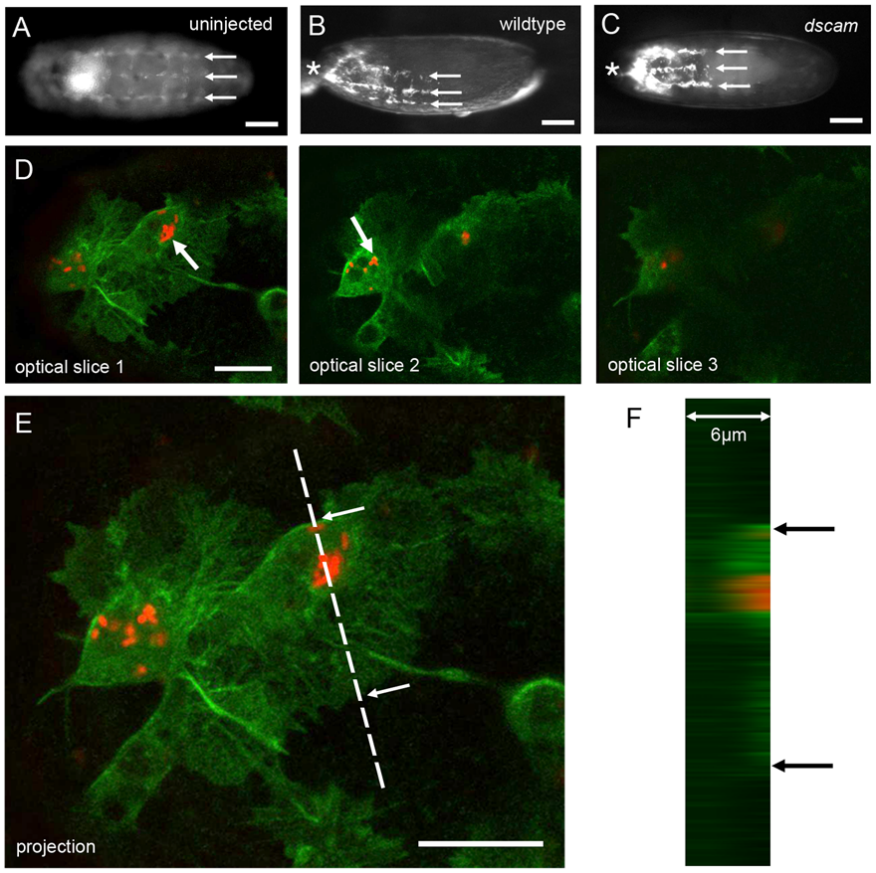
Injected *E. coli* are recognised and engulfed by embryonic hemocytes. (A) An embryo expressing GFP specifically in the hemocytes shows their characteristic pattern of distribution whereby the cells are arranged into three parallel lines running along the ventral surface of a stage 15 embryo (arrows). (B) Embryo injected with fluorescently labelled *E. coli* in the anterior region of the embryo (asterisk). 20 minutes after injection hemocytes become labelled as they bind the injected bacteria (arrows). (C) Hemocytes within *dscam^05518^* mutant embryos are able to recognize *E. coli* and can be seen to bind the fluorescently labelled bacteria (arrows) in a pattern indistinguishable from the wildtype. (D) Confocal images showing a series of optical slices taken through GFP expressing hemocytes. Images clearly show that the cells have internalised injected RFP labelled *E .coli* (arrows). (E) A projection of the slices shown in (D) highlight the two hemocytes (green) containing *E .coli* (red). (F) Z section taken through the region depicted by the dotted line on (E) clearly shows that the bacteria (red) are contained within the hemocyte (green). Arrows mark the cell extremities and correspond to the position of the arrows in (E). Scale bars represent 50 µm (A–C) and 10 µm (D and E).

To enable *in vivo* detection of *E. coli*, strain BL21 (DE3) was transformed with a high-copy vector pRSET expressing the monomeric red fluorescence protein. Protein expression was under the control of the T7 promoter; the leaky nature of this promoter allowed basal expression of the fluorescent protein without induction and successful detection of the bacteria within the embryo. The specificity of the bacterial-hemocyte interaction was initially tested by injecting stage 15 wild-type embryos containing unlabelled hemocytes with nl quantities of highly concentrated fluorescently-labelled bacterial suspension (10^10^ colony forming units/ml). At this embryonic stage, hemocytes are arranged into three characteristic lines that run anterior to posterior along the ventral aspect of the embryo (Figure 1A). Monitoring of the injected embryos under fluorescence revealed that 20 minutes after injection, bacteria specifically localised to the hemocytes. Thus, although the cells themselves were not fluorescently labelled their pattern of distribution could be easily visualised as a result of their binding to the RFP labelled bacteria alone (Figure 1B). The fact that the hemocytes can be seen in their normal positions within the embryo reveals that these cells do not have to actively migrate toward the invading bacteria but rather are able to recognize and bind the bacteria as they are washed over them in the extra-cellular space. To investigate this host-pathogen interaction in more detail hemocytes were labelled using the srpGAL4 driver to express GFP specifically in the hemocytes. These embryos, now with GFP labelled hemocytes, were then injected with a less concentrated fluorescently-labelled bacterial suspension (10^7^ cfu/ml) and subjected to timelapse confocal imaging. Confocal images show that hemocytes clearly locate and capture invading *E. coli* (Video S1) and optical sections collected at different focal planes through one hemocyte show labelled bacteria within the cytoplasm of the phagocyte (Figure 1D, E and F).

### Dscam-independent recognition of non-pathogenic *E. coli* by stage 15 *D. melanogaster* embryonic hemocytes

Previous work has indicated the importance of the immunoglobulin (Ig)-superfamily receptor Down syndrome cell adhesion molecule (Dscam) for bacterial recognition in *Drosophila* third instar larvae [20]. *Drosophila* fat body cells and hemocytes have the potential to express more than 36,000 isoforms of Dscam, and soluble isoforms of Dscam have also been detected in the hemolymph [20]. Dscam binds *E. coli* and is thought to act both as a phagocytic receptor and an opsonin in both *Drosophila* [20] and the malaria vector *Anopheles gambiae* [21]. To determine whether Dscam acts as a receptor for bacterial recognition in embryos, we compared the ability of hemocytes in wild-type and *dscam^05518^* mutant embryos [22] to bind *E. coli*. Surprisingly, in contrast to the above observations, *dscam^05518^* mutants were still able to recognise and crosslink bacteria on the surface of their hemocytes with equal efficiency to their wild-type counterparts demonstrating that Dscam is dispensable for recognition of *E. coli* by embryonic hemocytes (Figure 1C).

### *Photorhabdus* rapidly freezes embryonic hemocytes

**Figure 2:**
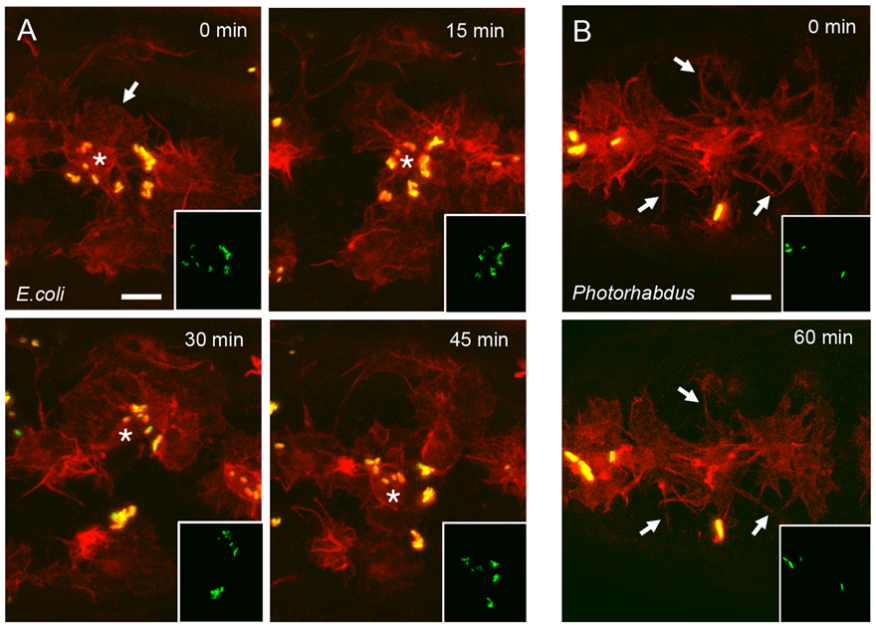
*Photorhabdus* injection causes a dramatic freezing of embryonic hemocytes. (A) Stills from a movie (see Video S2) of hemocytes expressing RFPmoesin following injection of GFP expressing *E. coli*. Hemocytes (asterisk) can be clearly seen actively migrating within the embryo and extending dynamic actin rich protrusions (arrow) as they bind and clear the injected *E. coli* (yellow). Insets show the movement of bacteria over the period of the movie as they are carried within the migrating hemocytes. (B) Stills from a movie (see Video S2) of RFP labelled hemocytes following injection of GFP labelled *Photorhabdus*. Hemocytes are able to recognise and bind the bacteria (yellow) but are completely frozen remaining in the same position for the duration of the movie (60 minutes). Cells still display actin rich protrusions (arrows) but these are static and bear no resemblance to the dynamic structures normally seen in hemocytes (compare to (A)). Insets show that the *Photorhabdus* bacteria themselves do not move throughout the movie as they are anchored to the static hemocytes. Scale bars represent 10 µm. Elapsed time is indicated in the upper right corner.

Having shown that embryonic hemocytes can bind and engulf live non-pathogenic bacteria, we wanted to characterize the response of embryos upon infection with an insect pathogen. *Photorhabdus* is a Gram-negative, nematode-vectored bacterium that kills a wide range of insect species [23]. Injection of stage 15 wild-type *Drosophila* embryos containing RFP-labelled hemocytes with a GFP-labelled *P. asymbiotica* suspension (10^7^ cfu/ml) had a profound effect on embryonic hemocyte motility whereby hemocytes rapidly loose their ability to migrate and apparently freeze (Figure 2 and Video S2). All actin rich protrusions are retained in these cells but appear to loose their dynamism failing to extend or retract as would ordinarily be seen in a healthy untreated motile hemocyte. This dramatic effect occurs very rapidly and could be observed 20 minutes after injection of *Photorhabdus*. Consequently the hemocytes are unable to engulf the bacteria. Interestingly, despite this severe effect on the cell's migratory and phagocytic machinery, their ability to recognize and bind the bacteria was unaffected (Figure 2B).

### Mcf1 causes rapid freezing of *Drosophila* embryonic hemocytes

**Figure 3:**
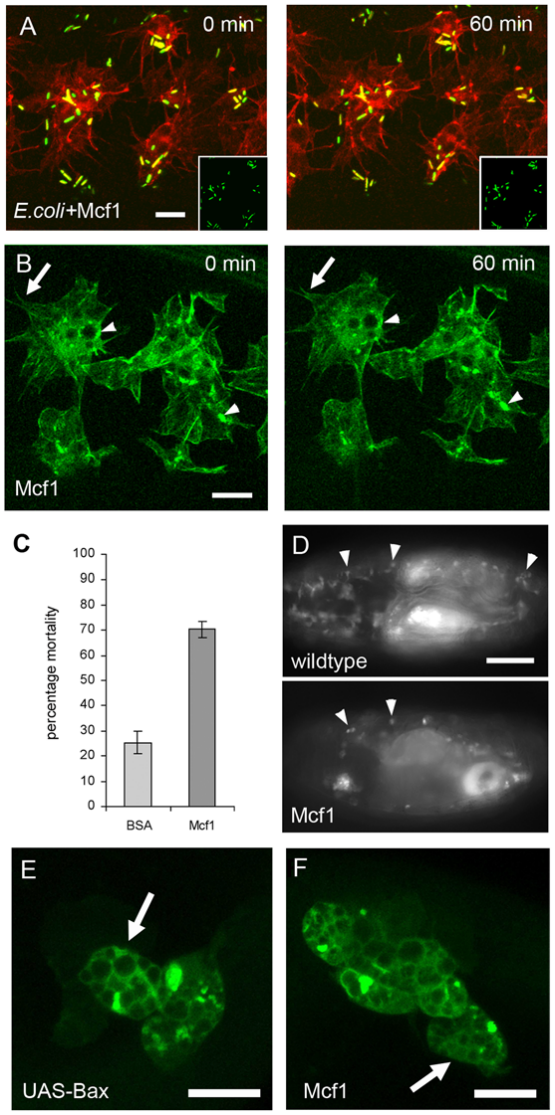
*E. coli* expressing Mcf1 are recognised by hemocytes and induce a freezing phenotype. (A) Stills from a movie (see Video S3) of hemocytes following injection of *E. coli* expressing Mcf1. The images clearly show that whilst the hemocytes can recognise the bacteria and bind them the cells are apparently frozen and fail to move throughout the whole 60 minutes of the movie. Insets show that the position of the bacteria does not change over the course of the movie. (B) Stills from a movie (see Video S4) of GFPmoesin expressing hemocytes following injection of purified Mcf1 show an identical effect to that seen in (A). Hemocytes are unable to move, have static actin rich protrusions (arrows) and over time accumulate GFP positive puncta in the cytoplasm (arrowheads). (C) Graph showing percentage mortality of embryos following Mcf1 injection. 75% of embryos fail to hatch following Mcf1 injection as opposed to 26% of those injected with heat inactivated BSA. (D) Embryos containing GFP expressing hemocytes (arrowheads) 12 hours after injection with Mcf1 (below) show a dramatic reduction in hemocyte number when compared with control embryos (above). (E) Hemocytes expressing UAS-Bax to induce apoptosis are morphologically distinct from hemocytes exposed to Mcf1 (compare with B) appearing circular and lacking any protrusive structures (arrow). (F) Confocal image of hemocytes within a dead embryo 12 hours after Mcf1 injection. Hemocytes appear identical to apoptotic cells expressing Bax (compare with E). Scale bars represent 10 µm (A, B, E and F) and 100 µm (D). Elapsed time is indicated in the upper right corner in A and B.

During insect infection *Photorhabdus* replicates within the hemocoel and delivers toxins which rapidly kill the insect host. Expression of one such toxin, Mcf1 is sufficient to allow *E. coli* to survive within, and kill, *Manduca* caterpillars which are otherwise able to clear *E. coli* infection [19]. Mcf1 treated caterpillars show rapid loss of body turgor (the “floppy” phenotype) within 12 hours, associated with massive apoptosis of the midgut epithelium. *Manduca* hemocytes also undergo apoptosis when exposed to recombinant Mcf1 [19]. Mammalian cells treated with Mcf1 also display key features of apoptosis which is putatively mediated by a BH3-like domain and involves the mitochondrial pathway [24],[25]. Injection of wild-type stage 15 embryos with *E. coli* constitutively expressing Mcf1 from the high-copy vector pUC18 causes rapid paralysis of embryonic hemocytes and inhibition of phagocytosis as observed following wild-type *Photorhabdus* infection (Figure 3A and Video S3). Micro-injection of purified Mcf1 (0.2 mg/ml) into wild-type stage 15 *Drosophila* embryos containing GFP-moesin expressing hemocytes also triggers rapid freezing of hemocytes with ‘frozen’ cellular protrusions and phagosomes (Figure 3B and Video S4). This effect was not seen when embryos were injected with the same concentration of heat inactivated BSA demonstrating that it is indeed the presence of Mcf1 that causes the freezing phenotype (Video S5). To ascertain whether the hemocyte paralysis effect of Mcf1 occurs in a dose-dependent manner we injected embryos with half the concentration previously used (0.1 mg/ml). In these embryos the freezing effect on hemocytes was less pronounced with many cells displaying active lamellipodial ruffling. Despite this however, these cells were not as dynamic as untreated cells and were still unable to migrate (Video S6). The rapid paralysis of hemocytes in the presence of Mcf1 suggests that this phenotype is independent, or upstream of, apoptosis given that the earliest pro-apoptotic indicators are observed 3 hours following incubation with Mcf1 [24]. To investigate this we expressed the pro-apoptotic Bcl-2 family member Bax in hemocytes using a combination of srpGAL4 and crqGAL4 drivers. Confocal analysis revealed that apoptotic hemocytes are very different in morphology to those exposed to Mcf1 appearing hugely swollen and containing fluorescent puncta having engulfed their dying neighbouring hemocytes (Figure 3E). Such obvious differences in morphology suggested that the early freezing effect of Mcf1 is independent of apoptosis but could not rule out a separate more long-term pro-apoptotic effect. In order to determine the long term effect of Mcf1 injection we left injected embryos overnight before scoring for lethality. We found that 70% of embryos injected with Mcf1 failed to develop to first larval instar compared with 26% of embryos injected with the same concentration of heat inactivated BSA (Figure 3C). This was consistent with a long-term apoptotic effect of Mcf1 causing widespread cell death and ultimately death of the animal. To investigate this further we observed hemocytes in embryos 12 hours after injection and found that hemocyte number is greatly reduced in these dead animals (Figure 3D). Additionally, high magnification confocal analysis revealed that the remaining hemocytes in these animals appeared morphologically identical to those overexpressing Bax (Figure 3F). This suggests that long-term exposure to Mcf1 causes hemocytes to ultimately undergo apoptosis consistent with previous studies using *Manduca* caterpillars.

**Figure 4:**
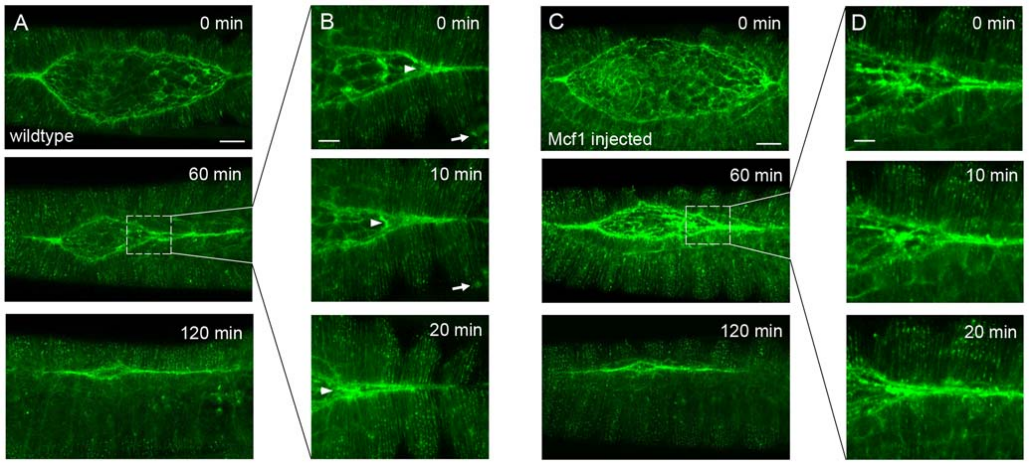
Mcf1 has no effect on dorsal closure. (A) Stills from a movie showing dorsal closure in an embryo expressing GFP moesin in all tissues. Over a period of 2 hours opposing lateral epithelial sheets sweep dorsally and fuse in the midline to seal the dorsal side of the embryo. (B) Stills from a movie (see Video S7) showing high magnification detail of the boxed region in A show that opposing epithelial fronts zipper together at the canthi (arrowhead) to close the dorsal hole. Arrows point to a hemocyte as it migrates beneath the epithelium. (C) Stills from a timelapse movie (see Video S7) of an embryo injected with Mcf1 show that the presence of the toxin has no effect on dorsal closure which proceeds at the same speed as observed in wildtype embryos (compare with (A)). (D) High magnification stills from zipping canthi show that epithelial fronts fuse as normal in these areas. Note that no dynamic hemocytes can be seen in these stills nor in the accompanying movie due to the freezing effect of Mcf1 on these cells. Scale bars represent 50 µm (A and C) and 10 µm (B and D). Elapsed time is indicated in the upper right corner.

We were curious to know whether the observed freezing effect of Mcf1 was specific for hemocytes or whether other cells might also be affected in the same way. To address this we analysed the paradigm morphogenetic tissue movement dorsal closure in embryos injected with Mcf1 and compared them to wildtype. Dorsal closure is a naturally occurring epithelial movement which requires the coordinated migration and fusion of two epithelial sheets to close the dorsal side of the embryo. Like hemocyte migration, dorsal closure requires the small GTPase Rac [26] and previous work has shown that the fusion of the migrating epithelial fronts is dependent on the formation of dynamic actin rich filopodia [27]. We found that Mcf1 injection into embryos expressing constitutively expressed GFP moesin had no effect on dorsal closure and that epithelial fronts migrated and fused at the same rate as wildtype (Figure 4 and Video S7). We therefore conclude that Mcf1 does not appear to affect all embryonic cell types in the dramatic fashion observed in hemocytes. One possible explanation for this result might be that epithelial cells are less endocytically active than hemocytes and therefore internalise less of the Mcf1 toxin.

### Cellular internalisation of Mcf1 is required for the freezing phenotype

**Figure 5:**
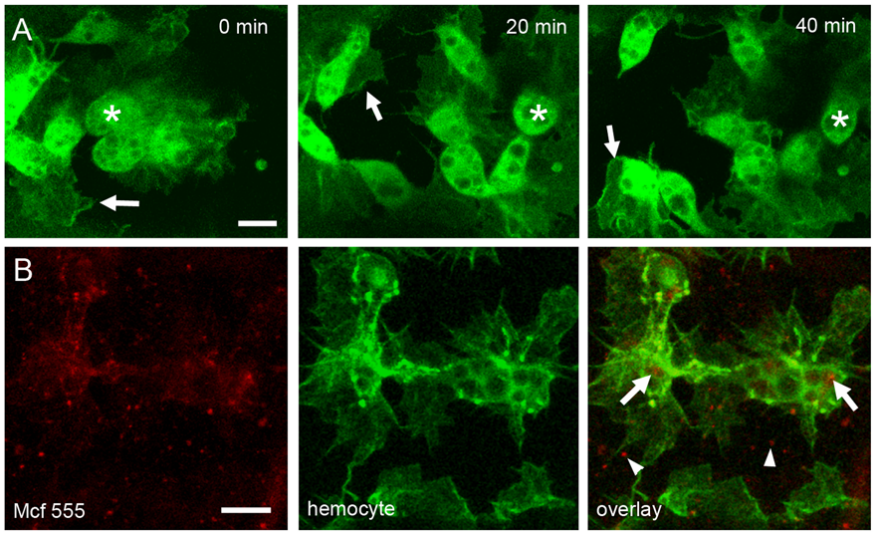
Internalisation of Mcf1 is required for freezing. (A) Stills from a timelapse movie (see Video S8) of hemocytes expressing *shibire^ts1^* following injection with 0.2 µg µl^−1^ Mcf1. Unlike wildtype cells, *shibire* mutant hemocytes do not freeze after exposure to Mcf1 and continue to migrate (asterisk) and extend large actin rich protrusions (arrows). Elapsed time is indicated in the upper right corner. (B) Injection of Alexa Fluro 555 labelled Mcf1 (Mcf1 555) into wildtype embryos shows localisation of labelled Mcf1 in hemocytes (white arrows) 1 hour after injection. Mcf1 can also be seen outside hemocytes indicating localisation in other surrounding cell types (arrowheads). Scale bars represent 10 µm.

Mcf1 has been previously described as requiring internalisation for cytotoxicity *in vitro* [24],[25]. To determine whether Mcf1 requires cellular internalisation for its freezing effect on hemocytes *in vivo* we tested whether *Drosophila* embryonic hemocytes attenuated in their ability to endocytose would still exhibit the freezing phenotype upon exposure to Mcf1. Dynamin is a GTP-binding protein which controls formation of constricted coat pits and is involved in a late step of clathrin-dependent endocytosis. In order to disrupt dynamin function specifically in hemocytes we expressed a temperature sensitive allele of *Drosophila* dynamin (*shibire^ts1^*) [28] using the a combination of the hemocyte drivers srpGAL4 and crqGAL4. We allowed embryos to develop to stage 14 before moving them to restrictive (non-permissive) temperature to allow activation of *shi^ts1^*. When these embryos were microinjected with Mcf1 the hemocytes were immune to the paralytic effect of the Mcf1 and continued to produce large dynamic cytoplasmic extensions appearing indistinguishable from uninjected control embryos (Figure 5A and Video S8). This demonstrates that the toxin Mcf1 needs to be internalized to cause cellular paralysis. To further investigate internalization and paralysis, Mcf1 was directly labelled with Alexa-Fluor 555 (Mcf1-555) and micro-injected into wild-type embryos. Embryos injected with Mcf1-555 show a punctate distribution of labelled Mcf1 within hemocytes appearing associated with phagosomal compartments (Figure 5B). Mcf1-555 was also visible outside of hemocytes in a similar punctate pattern probably due to internalization of the labelled toxin by cells other than the GFP expressing hemocytes since Mcf1 is known to affect a wide variety of cell types other than insect hemocytes [24].

### *Drosophila* embryos with disrupted Rac1 function evade Mcf1-mediated paralysis

**Figure 6:**
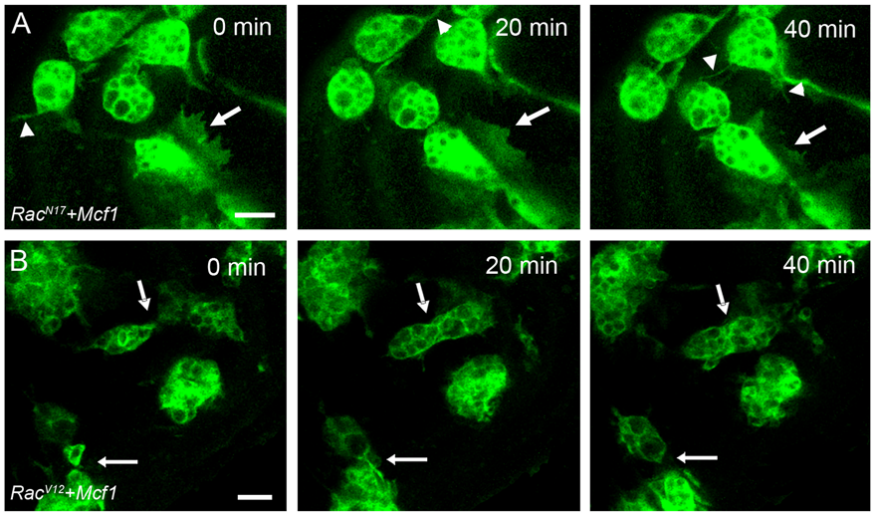
Embryos with defective Rac activity evade Mcf1 mediated paralysis. (A) Stills from a movie (see Video S9) of hemocytes expressing *Rac^N17^* following injection of Mcf1. Hemocytes expressing *Rac^N17^* are localised at the anterior of the embryo and have decreased lamellipodia formation and movement compared to wild-type cells. However, despite these defects *Rac^N17^* hemocytes fail to freeze after Mcf1 injection and continue to form small dynamic membrane ruffles (arrow) and filopodia (arrowheads). (B) Time-lapse movie stills (see Video S10) showing constitutively active *Rac^V12^* expressing hemocytes following Mcf1 injection. *Rac^V12^* expression in hemocytes causes reduced lamellipodia formation and migration when compared to wildtype cells. When exposed to Mcf1 these cells fail to display the freezing phenotype and like the *Rac^N17^* expressing cells continue to make small dynamic protrusions (arrows). Scale bars represent 10 µm. Elapsed time is indicated in the upper right corner.

The rapid onset of the freezing phenotype led to the hypothesis that Mcf1 may be acting on a pre-existing eukaryotic molecular switch governing actin cytoskeletal dynamics such as the rho GTPases. The small GTPase Rac is a key factor known to be involved in phagocytosis and cell migration in mammals and has been shown to be essential for hemocyte migration within the embryo [29],[30]. To investigate the potential involvement of Rac in Mcf1 mode of action we micro-injected Mcf1 into *Drosophila* embryos expressing either dominant- negative (Rac^N17^) or constitutively active (Rac^V12^) versions of the small GTPase, Rac, in hemocytes. It has been previously shown that hemocytes expressing dominant-negative Rac^N17^ fail to undergo their normal developmental migrations and exhibit stunted lamellipodia formation [29],[30]. However, despite these migratory defects, Rac^N17^ expressing hemocytes were completely resistant to the effects of Mcf1 and injection of Mcf1 into these embryos failed to cause the freezing effect observed when administered to wildtype cells (Figure 6A and Video S9). Interestingly, expression of constitutively active Rac^V12^ in hemocytes also led to complete resistance to Mcf1-mediated paralysis in the hemocytes (Figure 6B and Video S10). These results appear to indicate a role for Rac in Mcf1 mediated paralysis.

## Discussion

Adult *Drosophila* have been used extensively as infection models for a range of different microbes [1]. In this study, we have expanded this infection model to include the well studied *Drosophila* embryo. We have combined *Drosophila* genetics and real-time imaging to examine the very earliest stages of host-pathogen interaction, which are critical for the successful initiation of any infection. We have proven that embryonic hemocytes are indeed competent phagocytes when challenged with non-pathogenic *E. coli* and that the process of recognition and engulfment of these bacteria is, surprisingly, Dscam independent. In contrast, when injected with the insect and human pathogen *P. asymbiotica*, the highly motile embryonic hemocytes underwent a rapid paralysis, termed the ‘freezing’ phenotype. This phenotype could be phenocopied either by injection of the purified *Photorhabdus* toxin Mcf1 or by injection of recombinant *E. coli* expressing the *mcf1* gene. Use of *Drosophila* mutants either deficient in endocytic machinery or with altered activity of their Rac GTPases shows that the freezing phenotype requires internalization of the Mcf1 toxin and may involve unexpected alterations in the actin cytoskeleton of the hemocytes. These studies demonstrate not only that *Drosophila* embryos are powerful systems for studying the early stages of infection but also that they can facilitate the genetic dissection of the underlying molecular mechanisms of virulence and immunity.

Mcf1 is a single toxin which facilitates persistence of *Photorhabdus* bacteria in the hemocoel of an insect host in the face of the cellular immune response [19]. Previous studies have suggested that the massive apoptosis of the insect midgut epithelium, and destruction of insect hemocytes, associated with Mcf1 toxicity were related to its pro-apoptotic activity. However the rapid Mcf1 mediated hemocyte freezing phenotype described here suggests that this toxin may also have earlier effects on the actin cytoskeleton of host phagocytes. This early, anti-phagocytic, activity of Mcf1 may also be consistent with Mcf1 being the anti-phagocytic factor previously documented in other strains of *Photorhabdus* [31]. Mcf1 has previously been shown to require endocytosis for its pro-apoptotic activity and here we confirm that the freezing phenotype also requires internalisation of the toxin. The mechanism of how Mcf1 was freezing the actin cytoskeleton and preventing cellular migration was investigated by examining the effect of the toxin on embryonic hemocytes mutant in the small GTPase Rac. *Drosophila* embryonic hemocytes expressing dominant-negative or constitutively active Rac evaded the freezing phenotype caused by Mcf1 indicating a necessity for the presence of wild-type Rac in the freezing process. The Rho GTPases are a popular target for bacterial toxins as the manipulation of these molecules assists in virulence processes such as intracellular invasion and phagocytic avoidance [32]. A number of bacterial toxins inactivate Rho GTPases as a mechanism of avoiding phagocytosis. A group of such Rho inactivators act as Rho GTPase activating Proteins (RhoGAPs) which stimulate the intrinsic GTPase activity of the small GTPases hydrolysing them to their inactive GDP bound state. Examples of such toxins are ExoS and ExoT (*Pseudomonas aeruginosa*), YopE (*Yersinia* spp.) and SptP (*Salmonella typhimurium*) [33]–[36]. Constitutive activation of the Rho GTPases counteracts the activity of most GAP toxins and does not effect those that directly target the actin cytoskeleton [37],[38]. Whether Mcf1 is capable of inactivating Rac, and is doing so directly through a GAP-like activity or via other mechanisms remains to be explored.

Previous studies using third instar *Drosophila* larvae have implicated the immunoglobulin (Ig)-superfamily receptor Down syndrome cell adhesion molecule (Dscam) as being an important player in the recognition of bacteria [20]. Here we demonstrate that, despite these previous results, Dscam mutant hemocytes can recognize and bind *E. coli* with equal efficiency to that seen in wild-type embryos. This result demonstrates an intriguing difference between the immune system operating in the embryo when compared with larvae. Embryonic hemocytes are very long lived cells that persist into larval stages and constitute the circulating population of hemocytes in a larva. Within the larva a second population of hemocytes develops in a specialised hematopoetic organ called the lymph gland. Lymph gland hemocytes are normally released from this organ during metamporphosis but can be released prematurely following parasitisation [39]. Within an infected larvae, bacteria are therefore cleared by a combination of both embryonic hemocytes that have persisted through to larval stages and larval lymph gland hemocytes released upon infection. Our results suggest that the mechanisms used for bacterial recognition by these two populations could be different. We cannot exclude the possibility that hemocytes within the embryo operate with a small subset of the receptors utilised by lymph gland hemocytes and that as they persist through to larval stages they begin to express the full complement of immune receptors including Dscam. It will be interesting to determine whether this is the case or whether embryonic hemocytes encode a completely different set of proteins for bacterial detection. Further work is also needed to determine at which stage of development embryonic hemocytes acquire their ability to recognise invading micro-organisms.

The maturation of embryonic hemocytes as they progress through embryonic into larval stages of development is an interesting process that has received very little research attention. Recent studies have shown that when circulating within larvae, hemocytes appear substantially less motile than when they migrate throughout the embryo [40],[41] This difference in morphology can be attributed to their being passively pumped around the larval hemocoel rather than actively migrating through the embryonic extracellular space. Their morphology could change drastically however, once they encounter a pathogen that needs to be engulfed. Here we show the effect of Mcf1 on the actin cytoskeleton of a hemocyte within an embryo which manifests itself as a block on cell migration. It would be interesting to see the affect of Mcf1 on hemocytes within larvae where its effect on cell migration would presumably be less pronounced but its effect on other actin dependent processes such as phagocytosis may be equally drastic.

Here we have described an in vivo system for looking at a known population of phagocytes in a closed system, the *Drosophila* embryo. This system complements the use of tissue culture systems for several reasons. First, the real-time behaviour of phagocytes in their natural environment can be monitored. This overcomes the limitations of looking at immortal cell lines of uncertain origin (eg Schneider cells) or of looking at abnormal behaviour in primary cultures of phagocytes (eg hemocytes recently bled from an insect). Second, we can use both genetic mutants and RNAi to look at effects in vivo. This contrasts to transfection experiments on cell cultures that are often transient and variable in their effects. Finally, although we cant precisely define the concentration of effector proteins delivered into the hemocyte via injection, we can say, in the case of Mcf1, that both the purified protein, the recombinant protein expressed by *E. coli* and the native Mcf1 expressed by *P. asymbiotica* all had the same phenotypic effects on the in vivo system. Moreover, these effects were all very different to those previously described for Mcf1 protein applied to primary cultures of *Manduca* hemocytes recorded under time-lapse photography [19].

In recent years cultured *Drosophila* S2 cells have been used extensively as a model system to study infection and immune responses. These cells allow for large scale screening using RNAi and have been successfully used to identify proteins involved in host interactions with important human bacterial pathogens such as *Escherichia coli*, *Staphylococcus aureus* [42],[43], *Mycobacterium spp.* [44], *Legionella pneumophila* [45], *Chlamydia spp*. [46]–[48] and *Listeria monocytogenes* [49]–[51]. Whilst such studies provide a reservoir of genes involved in bacterial recognition and degradation *in vitro* the situation *in vivo* where hemocytes can interact with other immune cells to optimize immune responses is likely to be more complex. Ultimately, to have an impact on human and animal health, the results obtained by *in vitro* studies need to be verified in a whole organism. The assay we present here provides a perfect model to begin to fill these gaps and should lead to a better understanding of host-pathogen interactions in the complex setting of a multicellular organism.

## Materials and Methods

### Bacterial strains and plasmids

*P. asymbiotica* ATCC 43949 was isolated from a human leg wound in San Antonio, Texas [52] and obtained from the ATCC culture collection. A spontaneous rifampicin mutant was created by common microbiology methods and used in all microinjection experiments. *Escherichia coli* S17-1λpir [53] was used as a conjugative donor of the *pir*-dependent suicide replicon pBamH7 (a kind gift from Dr Leo Erbel) which constitutively expresses green fluorescence protein (GFP). *E. coli* BL21 was used for cloning and constitutive expression of Mcf1 from pUC18 (as previously described [19]) and green or red fluorescence protein (RFP) from pRSET (Invitrogen). DNA fragments were cloned using standard cloning procedures. Bacterial strains were amplified in LB broth containing, as appropriate, ampicillin 100 µg ml^−1^; kanamycin 25 µg ml^−1^; rifampicin 25 µg ml^−1^. For embryo microinjections, bacteria were grown to stationary phase at 37°C for 18–24 h, washed in phosphate-buffered saline (PBS) and adjusted to the appropriate density.

### Plasmid conjugation to *P. asymbiotica*

pBamH7 was delivered to *P. asymbiotica* via conjugation with S17-1λpir by using a membrane filter mating technique. S17-1λpir pBamH7 was inoculated into 5 ml of LB broth containing kanamycin and grown at 37°C for 16–18 h with shaking (200 rpm). *P. asymbiotica* was grown at 28°C for 16–18 h with shaking (200 rpm) but without antibiotic selection. 100 µl of each saturated bacterial culture was added to 3 ml of sterile 10 mM MgSO_4_, mixed, and filtered through a 0.45-µm-pore-size nitrocellulose filter, using a 25-mm Swinnex filter apparatus (Millipore). Control assays, using donor and recipient alone, were also performed. Filters were placed on LB plates supplemented with 10 mM MgSO_4_ and incubated for at least 8 h in a 37°C incubator. The filters were washed with 4 ml of sterile 0.85% NaCl, and 100 µl aliquots were spread onto LB plates containing 25 µg of rifampicin and 25 µg of kanamycin per ml. Rifampicin-resistant and kanamycin-resistant transconjugants were identified after 48 h incubation at 37°C.

### Preparation of Mcf1 for micro-injection

Purification and labelling of Mcf1 was carried out as described previously [24]. Mcf1 was diluted to required concentrations for micro-injection with sterile 1× phosphate buffered saline solution (PBS).

### Fly stocks

UAS constructs were expressed in hemocytes using either the hemocyte specific Gal4 line *serpentHemoGAL4* (*srpGAL4*; [54]) or *croquemortGAL4* (*crqGAL4*; [30]. A *w; srpGAL4, UAS-GFP* recombinant line was used to visualize wildtype hemocyte motility and bacterial engulfment. Actin dynamics were visualised in hemocytes using lines with recombined chromosomes carrying both *srpGAL4* and either *UASGFPmoesin* (*UASGMA*; [55] or *UAS-RFP-Moesin* [56]. Embryos containing the transgene sGMCA (constitutively expressing GFP-Moesin) [55] were used to visualise actin dynamics in epithelial cells during dorsal closure. To activate apoptosis in hemocytes *w;srpHemoGAL4UASGFP;crqGAL4UASGFP* flies were crossed to a *w;UAS-bax* stock. After egg laying at 25°C, embryos were transferred to 29°C to develop. To disrupt *shibire* function in hemocytes *w;srpHemoGAL4UASGFP;crqGAL4UASGFP* flies were crossed to a *w;UASshi^ts1^* stock [28] generating *w;srpHemoGAL4UASGFP/UASshi^ts1^; crqGAL4UASGFP/+* progeny. These embryos were then left to develop to late stage 13 before being incubated at 32°C for 2 h and returning to room temperature for 1.5 h before injection. Expression of dominant negative Rac constructs in hemocytes was achieved by crossing *UAS-Rac^V12^* or *UAS-Rac^N17^* flies to a *w;srpHemoGAL4UASGFP;crqGAL4UASGFP* stock. For the Dscam loss of function experiment the *dscam^05518^* allele was used [22]. *w; dscam/CTG* flies were intercrossed and homozygous *dscam^05518^* mutants were identified by their lack of a fluorescent balancer.

### Micro-injection and imaging of *Drosophila* embryos

Embryos were collected at stage 15 of development and prepared for micro-injection and confocal imaging. Embryos were dechorionated in bleach and mounted on a coverslip under Voltalef oil as previously described [57]. Micro-injection was carried out using an Eppendorf Femtojet injectman. The micro-injection needle was loaded with 4 µl of either *E. coli RFP*, *E. coli GFP pUC-18*, *GFP pUC-18mcf1* or purified Mcf1 for injection as required. The needle was then introduced into the anterior of the embryo and the embryo injected. Following injection a coverslip was mounted over the embryos ready for microscopy. For the survival study injected embryos were left uncovered in voltalef oil in a humid chamber overnight and scored for lethality the following day.

Imaging was carried out on a Zeiss LSM-510 confocal laser-scanning microscope (Zeiss LSM-510 system with inverted Axiovert 100 M microscope), equipped with a krypton-argon laser and helium-neon lasers, under 63× objective. For time-lapse movies images were obtained by taking four optical slices (each slice averaged 2×) through hemocytes collected at 120 s intervals. Compilation and processing of movies was carried out using ImageJ software.

## Supporting Information

Video S1 Embryonic hemocytes engulf *E. coli*. Confocal timelapse movie of an embryo containing GFP expressing hemocytes (green) that has been subjected to injection of RFP expressing *E. coli* (red). In the first few frames of the movie, the bacteria are rapidly engulfed by a hemocyte. Movie duration is 50 minutes.
(1.59 MB MOV)

Video S2 *Photorhabdus* rapidly freezes embryonic hemocytes. The movie on the left shows an embryo containing RFP expressing hemocytes (red) that has been injected with *E. coli* (yellow). The hemocytes are highly motile cells migrating within the embryo as they clear the invading bacteria. The movie on the right shows the effect of injection of *Photorhadus* (yellow) on the hemocytes (red). Hemocytes are able to bind the bacteria but appear frozen and lose their ability to move. Movie duration is 40 minutes. Both movies are running at the same speed (7 fps).
(2.42 MB MOV)

Video S3 Mcf1 causes freezing of hemocytes. Confocal timelapse movie showing embryonic hemocytes expressing RFP in an embryo injected with GFP expressing *E. coli* producing Mcf1. Top panel shows the red channel (RFP), the middle shows the green channel (GFP) and the lower is the merge. Whilst the hemocytes (red) are able to detect the bacteria (green) they are unable to move and appear completely frozen as seen following injection with *Photorhabdus* (compare with Video S2). Movie duration is 50 minutes.
(1.07 MB MOV)

Video S4 Injection of purified Mcf1 causes hemocyte freezing. Movie of GFPmoesin expressing hemocytes within an embryo after injection with 0.2 mg/ml purified Mcf1. Hemocytes are able to form actin rich protrusions but these protrusions are completely static and bear no resemblance to the highly dynamic structures normally seen in these cells (compare to Video S1). Movie duration is 60 minutes.
(1.97 MB MOV)

Video S5 Injection of BSA has no effect on hemocyte dynamics. Movie of GFPmoesin expressing hemocytes within an embryo following injection with 0.2 mg/ml heat inactivated BSA. Hemocytes appear completely wildtype in their morphology and actin dynamics extending large lamellipodia as they patrol their environment.
(0.23 MB MOV)

Video S6 Hemocyte paralysis effect of Mcf1 occurs in a dose-dependent manner. Movie of GFPmoesin expressing hemocytes within an embryo following injection with 0.1 mg/ml Mcf1. The freezing effect on hemocytes is less pronounced at the lower concentration (compare with Video S4) with many cells displaying active lamellipodial ruffling although these cells are not as dynamic as untreated cells and were still unable to migrate.
(0.93 MB MOV)

Video S7 Mcf1 has no effect on dorsal closure. Movie showing dorsal closure in wildtype embryos (left) and embryos injected with Mcf1 (right). Both embryos express sGMCA (constitutively expressed GFPmoesin) to enable epithelial dynamics to be visualised. Epithelial fronts migrate and fuse at the same rate in both embryos demonstrating that Mcf1 has no effect on dorsal closure. Arrow in the wildtype image highlights a dynamic hemocyte as it migrates under the epithelium. No migrating hemocytes can be seen in the Mcf1 treated embryo due to the specific freezing effect on these cells.
(2.45 MB MOV)

Video S8 Cellular internalisation of Mcf1 is required for hemocyte freezing. Timelapse confocal movie showing embryos containing hemocytes expressing *shibire^ts1^* injected with Mcf1. These *shibire* mutant hemocytes can be seen migrating normally extending large, dynamic actin rich protrusions and show no sign of the freezing phenotype observed in wildtype cells upon exposure to Mcf1 toxin. Movie duration is 40 minutes.
(1.95 MB MOV)

Video S9 Mcf1 induced freezing is blocked in Rac^N17^ expressing cells. Movie showing Rac^N17^ expressing hemocytes in an embryo injected with Mcf1. Due to a requirement of Rac for the formation of lamellipodia, these mutant cells are far less mobile than wildtype hemocytes and produce much smaller and less dynamic protrusions. However, despite these defects the cells are still motile after exposure to Mcf1 and there is no evidence of the cell freezing effect observed in wildtype cells (compare to Videos S2, S3 and S5). Movie duration is 60 minutes.
(2.59 MB MOV)

Video S10 Rac^V12^ expression in hemocytes also blocks freezing effect of Mcf1. Timelapse movie showing Rac^V12^ expressing hemocytes in an embryo injected with Mcf1. Like Rac^N17^, expression of Rac^V12^ causes migration defects in hemocytes. Despite this when exposed to Mcf1, the mutant cells appear immune to the paralytic effect of the toxin. Movie duration is 60 minutes.
(2.52 MB MOV)

## References

[1] Vallet-Gely I, Lemaitre B, Boccard F (2008). Bacterial strategies to overcome insect defences.. Nat Rev Microbiol.

[2] Hedges LM, Johnson KN (2008). Induction of host defence responses by Drosophila C virus.. J Gen Virol.

[3] Chamilos G, Lewis RE, Hu J, Xiao L, Zal T (2008). Drosophila melanogaster as a model host to dissect the immunopathogenesis of zygomycosis.. Proc Natl Acad Sci U S A.

[4] Nehme NT, Liegeois S, Kele B, Giammarinaro P, Pradel E (2007). A model of bacterial intestinal infections in Drosophila melanogaster.. PLoS Pathog.

[5] Shirasu-Hiza MM, Schneider DS (2007). Confronting physiology: how do infected flies die?. Cell Microbiol.

[6] Tepass U, Fessler LI, Aziz A, Hartenstein V (1994). Embryonic origin of hemocytes and their relationship to cell death in Drosophila.. Development.

[7] Wood W, Faria C, Jacinto A (2006). Distinct mechanisms regulate hemocyte chemotaxis during development and wound healing in Drosophila melanogaster.. J Cell Biol.

[8] Wood W, Jacinto A (2007). Drosophila melanogaster embryonic haemocytes: masters of multitasking.. Nat Rev Mol Cell Biol.

[9] Olofsson B, Page DT (2005). Condensation of the central nervous system in embryonic Drosophila is inhibited by blocking hemocyte migration or neural activity.. Dev Biol.

[10] Pielage JF, Powell KR, Kalman D, Engel JN (2008). RNAi screen reveals an Abl kinase-dependent host cell pathway involved in Pseudomonas aeruginosa intenalization.. PLoS Pathog.

[11] Kim SH, Park SY, Heo YJ, Cho YH (2008). Drosophila melanogaster-based screening for multihost virulence factors of Pseudomonas aeruginosa PA14 and identification of a virulence-attenuating factor, HudA.. Infect Immun.

[12] Frandsen JL, Gunn B, Muratoglu S, Fossett N, Newfeld SJ (2008). Salmonella pathogenesis reveals that BMP signaling regulates blood cell homeostasis and immune responses in Drosophila.. Proc Natl Acad Sci U S A.

[13] Brandt SM, Dionne MS, Khush RS, Pham LN, Vigdal TJ (2004). Secreted Bacterial Effectors and Host-Produced Eiger/TNF Drive Death in aSalmonella-Infected Fruit Fly.. PLoS Biol.

[14] Blow NS, Salomon RN, Garrity K, Reveillaud I, Kopin A (2005). Vibrio cholerae infection of Drosophila melanogaster mimics the human disease cholera.. PLoS Pathog.

[15] Fleming V, Feil E, Sewell AK, Day N, Buckling A (2006). Agr interference between clinical Staphylococcus aureus strains in an insect model of virulence.. J Bacteriol.

[16] Gerrard J, Waterfield N, Vohra R, ffrench-Constant R (2004). Human infection with *Photorhabdus asymbiotica*: an emerging bacterial pathogen.. Microbes and Infection.

[17] Gerrard JG, Joyce SA, Clarke DJ, ffrench-Constant RH, Nimmo GR (2006). Nematode symbiont for *Photorhabdus asymbiotica*.. Emerg Infect Dis.

[18] Waterfield NR, Sanchez-Contreras M, Eleftherianos I, Dowling A, Wilkinson P (2008). Rapid Virulence Annotation (RVA): identification of virulence factors using a bacterial genome library and multiple invertebrate hosts.. Proc Natl Acad Sci.

[19] Daborn PJ, Waterfield N, Silva CP, Au CP, Sharma S (2002). A single Photorhabdus gene, makes caterpillars floppy (mcf), allows Escherichia coli to persist within and kill insects.. Proc Natl Acad Sci U S A.

[20] Watson FL, Puttmann-Holgado R, Thomas F, Lamar DL, Hughes M (2005). Extensive diversity of Ig-superfamily proteins in the immune system of insects.. Science.

[21] Dong Y, Taylor HE, Dimopoulos G (2006). AgDscam, a hypervariable immunoglobulin domain-containing receptor of the Anopheles gambiae innate immune system.. PLoS Biol.

[22] Schmucker D, Clemens JC, Shu H, Worby CA, Xiao J (2000). Drosophila Dscam is an axon guidance receptor exhibiting extraordinary molecular diversity.. Cell.

[23] Clarke DJ (2008). *Photorhabdus*: a model for the analysis of pathogenicity and mutualism.. Cell Microbiol.

[24] Dowling AJ, Waterfield NR, Hares MC, Le Goff G, Streuli CH (2007). The Mcf1 toxin induces apoptosis via the mitochondrial pathway and apoptosis is attenuated by mutation of the BH3-like domain.. Cell Microbiol.

[25] Dowling AJ, Daborn PJ, Waterfield NR, Wang P, Streuli CH (2004). The insecticidal toxin Makes caterpillars floppy (Mcf) promotes apoptosis in mammalian cells.. Cell Microbiol.

[26] Woolner S, Jacinto A, Martin P (2005). The small GTPase Rac plays multiple roles in epithelial sheet fusion–dynamic studies of Drosophila dorsal closure.. Dev Biol.

[27] Jacinto A, Wood W, Balayo T, Turmaine M, Martinez-Arias A (2000). Dynamic actin-based epithelial adhesion and cell matching during Drosophila dorsal closure.. Curr Biol.

[28] Kitamoto T (2001). Conditional modification of behaviour in Drosophila by targeted expression of a temperature-sensitive shibire allele in defined neurons.. J Neurobiology.

[29] Paladi M, Tepass U (2004). Function of Rho GFPases in embryonic blood cell migration in *Drosophila*.. J Cell Sci.

[30] Stramer B, Wood W, Galko MJ, Redd MJ, Jacinto A (2005). Live imaging of wound inflammation in Drosophila embryos reveals key roles for small GTPases during in vivo cell migration.. J Cell Biol.

[31] Silva CP, Waterfield NR, Daborn PJ, Dean P, Chilver T (2002). Bacterial infection of a model insect: *Photorhabdus luminescens* and *Manduca sexta*.. Cellular Microbiology.

[32] Orth JH, Lang S, Taniguchi M, Aktories K (2005). Pasteurella multocida toxin-induced activation of RhoA is mediated via two families of G(alpha} proteins, G(alpha}q and G(alpha}12/13.. J Biol Chem.

[33] Black DS, Bliska JB (2000). The RhoGAP activity of the Yersinia pseudotuberculosis cytotoxin YopE is required for antiphagocytic function and virulence.. Mol Microbiol.

[34] Goehring UM, Schmidt G, Pederson KJ, Aktories K, Barbieri JT (1999). The N-terminal domain of Pseudomonas aeruginosa exoenzyme S is a GTPase-activating protein for Rho GTPases.. J Biol Chem.

[35] Krall R, Schmidt G, Aktories K, Barbieri JT (2000). Pseudomonas aeruginosa ExoT is a Rho GTPase-activating protein.. Infect Immun.

[36] Fu Y, Galán JE (1999). A salmonella protein antagonizes Rac-1 and Cdc42 to mediate host-cell recovery after bacterial invasion.. Nature.

[37] Fiorentini C, Donelli G, Matarrese P, Fabbri A, Paradisi S (1995). Escherichia coli cytotoxic necrotizing factor 1: evidence for induction of actin assembly by constitutive activation of the p21 Rho GTPase.. Infect Immun.

[38] Sheahan KL, Satchell KJ (2007). Inactivation of small Rho GTPases by the multifunctional RTX toxin from Vibrio cholerae.. Cell Microbiol.

[39] Lanot R, Zachary D, Holder F, Meister M (2001). Postembryonic hematopoiesis in Drosophila.. Dev Biol.

[40] Babcock DT, Brock AR, Fish GS, Wang Y, Perrin L (2008). Circulating blood cells function as a surveillance system for damaged tissue in Drosophila larvae.. Proc Natl Acad Sci.

[41] Pastor-Pareja JC, Wu M, Xu T (2008). An innate immune response of blood cells to tumors and tissue damage in Drosophila.. Dis Model Mech.

[42] Stuart LM, Deng J, Silver JM, Takayashi K, Tseng AA (2005). Response to Staphylococcus aureus requires CD36-mediated phagocytosis triggered by the COOH-terminal cytoplasmic domain.. J Cell Biol.

[43] Ramet M, Manfruelli P, Pearson A, Mathey-Prevot B, Ezekowitz RA (2002). Functional genomic analysis of phagocytosis and identification of a Drosophila receptor for E. coli.. Nature.

[44] Philips JA, Rubin EJ, Perrimon N (2005). Drosophila RNAi screen reveals CD36 family member required for mycobacterial infection.. Science.

[45] Dorer MS, Kirton D, Bader JS, Isberg RR (2006). RNA interference analysis of Legionella in Drosophila cells: exploitation of early secretory apparatus dynamics.. PLoS Pathog.

[46] Elwell CA, Ceesay A, Kim JH, Kalman D, Engel JD (2008). RNA interference screen identifies Abl kinase and PDGFR signalling in Chlamydia trachomatis entry.. PLoS Pathog.

[47] Elwell C, Engel JN (2005). Drosophila melanogaster S2 cells: a model system to study Chlamidia interaction with host cells.. Cell Microbiol.

[48] Derre I, Pypaert M, Dautry-Varsat A, Agaisse H (2007). RNAi screen in Drosophila cells reveals the involvement of the Tom complex in Chlamydia infection.. PLoS Pathog.

[49] Cheng LW, Viala JP, Stuurman N, Wiedemann U, Vale RD (2005). Use of RNA interference in Drosophila S2 cells to identify host pathways controlling compartmentalization of an intracellular pathogen.. Proc Natl Acad Sci U S A.

[50] Cheng LW, Portnoy DA (2003). Drosophila S2 cells: an alternative infection model for Listeria monocytogenes.. Cell Microbiol.

[51] Agaisse H, Burrack LS, Philips JA, Rubin EJ, Perrimon N (2005). Genome-wide RNAi screen for host factors required for intracellular bacterial infection.. Science.

[52] Farmer JJd, Jorgensen JH, Grimont PA, Akhurst RJ, Poinar GO (1989). Xenorhabdus luminescens (DNA hybridization group 5) from human clinical specimens.. J Clin Microbiol.

[53] Simon R, Preifer U, Puhler A (1983). A broad host range mobilisation system for in vivo genetic engineering: transposon mutagenesis in Gram-negative bacteria.. Bio/Technology.

[54] Bruckner K, Kockel L, Duchek P, Luque CM, Rorth P (2004). The PDGF/VEGF receptor controls blood cell survival in Drosophila.. Dev Cell.

[55] Kiehart DP, Galbraith CG, Edwards KA, Rickoll WL, Montague RA (2000). Multiple forces contribute to cell sheet morphogenesis for dorsal closure in Drosophila.. J Cell Biol.

[56] Millard T, Martin P (2008). Dynamic analysis of filopodial interactions during the zippering phase of Drosophila dorsal closure.. Development.

[57] Wood W, Jacinto A, Guan J-L (2004). Imaging Cell Movement During Dorsal Closure in Drosophila Embryos.. Methods in Molecular Biology, vol294: Cell Migration: Developmental Methods and Protocols.

